# Labor market entry prospects of people with visual impairments: evidence from administrative register data in the Netherlands

**DOI:** 10.1007/s10198-025-01830-2

**Published:** 2025-09-15

**Authors:** Katharina Diehl, Eline Heppe, Melvin Vooren, Ilja Cornelisz, Chris van Klaveren

**Affiliations:** 1 Amsterdam Center for Learning Analytics, Faculty of Behavioral and Movement Sciences, Vrije Universiteit Amsterdam, van der Boechorstraat 7, Amsterdam, Netherlands; 2 Kentalis Academy, Royal Kentalis, Utrecht, Netherlands

**Keywords:** Visual impairment, School-to-work transition, Socio-economic outcomes, Labor market outcomes, Matching, Duration model, Competing risks survival analysis

## Abstract

The UN’s Convention on the Rights of Persons with Disabilities includes a right to equal work opportunities. People with visual impairments (VI) are targeted by this convention and experience low employment rates. The initial transition from education to labor is a predictor for later labor market participation. Exploiting administrative register data, we identified a graduated subpopulation with VI and matched them to a control group. This paper evaluates (1) group differences in various socio-economic outcomes following graduation, and (2) following initial employment. Following graduation there are fewer transitions into (self-)employment for people with VI and more transitions to disability insurance or social benefits, but similar patterns for unemployment insurance and further education. Scrutinizing subsequent dynamics for those that secured initial employment revealed a greater share of those with VI maintained employment as dominant source of income given there was a change in employment status. However, there are more dynamics recorded for individuals with VI, which may reflect difficulties retaining a job. This group remained overrepresented in disability insurance as well as social benefits. Differences with regards to self-employment, unemployment insurance, further education and the other no income status did not reach significance. In conclusion, this study did not find equal labor market prospects for declarants with VI. The finding that VI declarants face less favorable labor market prospects than their matched counterparts, despite having similar cognitive ability, underscores the need for policies that not only improve their labor market outcomes but also promote greater equality in opportunities to reach the UN convention’s ideals.

**Supplementary Information:**

The online version contains supplementary material available at 10.1007/s10198-025-01830-2.

## Introduction

The United Nations drafted the Convention on the Rights of Persons with Disabilities in 2006, explicitly stating the right of people with disabilities to participate freely and fully in the labor market [1]. The Netherlands ratified this convention in 2016 [2]. This might not only benefit people with disabilities [3], but also governments, as increased paid employment among disabled persons reduces social welfare benefit payments and increases tax revenues. This study empirically examines whether observed labor market entry patterns for people with visual impairments (VI) who made an insurance claim for sensory disability care in the Netherlands (hereafter referred to as declarants with VI) are similar to a matched control group with otherwise comparable observable characteristics. Thus, we aim to verify if people with VI in the Netherlands can enter freely and fully in the labor market.

Dutch policies aimed at removing labor market barriers and helping people find jobs are generally targeted at individuals with disabilities rather than specifically at those with VI. For instance, the Participation Act (In Dutch: Participatiewet) was implemented in 2015. This nationwide welfare reform aims to promote the re-employment of unemployed workers and help people with disabilities find employment. An evaluation shows that this reform was rather unsuccessful in the first five years [4]. An analysis based on vocational rehabilitation data from the US seemed to corroborate the challenges associated with increasing labor force participation among people with VI. The impact of certain vocational rehabilitation services was found to be minimal or even counterproductive [5]. Meanwhile, extensive research indicates that (young) people with VI have a higher probability of being unemployed [6–10]. Studies evaluating the labor market participation of people with VI typically rely on cross-sectional surveys, which do not always include a control group. These studies consistently report low employment rates among people with VI and, when a control group is included, significant discrepancies in labor market participation between people with VI and the control group. For example, survey studies report employment rates of 39.3%, 40.8%, 49%, and 77% for different VI samples, compared to 76.6% and 88.8% for controls groups [8, 11–13]. Additionally, there is empirical evidence showing that a successful school-to-work transition can have positive and long-lasting effects [14, 15]. Although, to our knowledge, there are not yet studies exploring the school-to-work transition among graduates with VI. Consequently, this study specifically focuses on the school-to-work transition after graduation for people with VI in the Netherlands, using administrative register data and comparing them to a matched control group.

More specifically, this study contributes in three ways. First, using duration models accounting for competing risks, it compares the labor market dynamics of declarants with VI and a matched control group by focusing on a variety of socio-economic outcomes (SEOs) such as (un)employment, self-employment, further education, and receiving social welfare benefits or insurance. To ensure that the declarants with VI and the control group have comparable background characteristics at the time of graduation, a statistical matching approach is used. Second, recognizing that a successful school-to-work transition is not guaranteed by merely securing a job upon graduation, the study examines the stability of initial employment. A well-established empirical finding is that individuals often achieve initial employment but experience only short periods of employment, repeatedly alternating between job seeking and low-level employment [16]. Furthermore, individuals with disabilities are, on average, three times more likely to drop out of initial employment [17]. Therefore, this study also compares the labor market dynamics of declarants with VI and a matched control group after initially securing a job upon graduation. Again, a statistical matching approach is used to ensure that the declarants with VI and the control group have comparable background characteristics at the moment of securing initial employment. Third, we explore potential secondary sources of income among both groups.

For the analyses individual-level administrative register data from SN is used. These data combine registers from a variety of governmental and non-governmental sources, including municipal registration data and registration data related to graduation, welfare, employment, healthcare, and insurance. A subpopulation of declarants with VI in the Netherlands was established based on health insurance records from 2015 to 2019. Because this study focuses on the school-to-work transition, only individuals with a graduation date from vocational or higher education between 1999 and 2019 were selected.

The analyses were conducted on declarants with VI, identifying a total of 2,412 individuals. To construct their matched counterparts, we extracted a random sample of 2 million people from the Dutch general population and selected only those who graduated from vocational or higher education between 1999 and 2019 and did not claim sensory disability care in the Netherlands to their health insurance. This resulted in a subsample of 309,176 individuals, which formed the basis for constructing a credible control group via a nearest neighbor statistical matching procedure.

When focusing on labor market dynamics after initially securing a job upon graduation, we restrict the analysis to individuals who secured initial employment after graduation. This leaves a group of 1,252 declarants with VI (51.9%) and a subsample of 202,126 people (65.4%) from the Dutch general population. This subsample is used to construct the matched control group through a nearest neighbor statistical matching procedure. Competing risks regression models were performed to examine the differences in SEOs observed after graduation and after initial employment.

The advantage of using administrative register data is that it circumvents issues of selection and response biases commonly encountered in survey-based research [18]. While declarants with VI may not represent all people with VI in the Netherlands, the use of administrative register ensures that diagnostic information is not dependent on self-reports or ambiguous criteria for a large group of people with VI. Furthermore, the longitudinal nature of administrative register data allows us to explore dynamics SEOs throughout the first two transitions post-graduation.

Additionally, the administrative register data provides a comprehensive overview of various SEOs beyond the binary employed/unemployed status. This allows for a better insight into the different sources through which both the employed and unemployed are deriving their income and allocating their time. The data used in this study also allow for the identification of graduates with VI who are neither employed nor engaged in education or training (the so-called NEETs). Not only can we explore details on their sources of income, such as unemployment or social benefits, but also isolate a group who does not derive any income from any of the various SEOs. Additionally, the longitudinal nature of this type of data allows us to observe the time dynamics surrounding the initial transition upon graduation and to evaluate subsequent dynamics upon finding initial employment. This is also particularly valuable for policymakers concerned with the labor market prospects of vulnerable groups, including those with VI. Furthermore, the data also enables us to observe and account for the potential influence of timing differences on the probability of observing certain labor market transitions.

This study proceeds as follows. Section 2, discusses the matching and duration model methodologies used. Section 3, describes the Dutch administrative register data used in the analysis. Section 4 presents the empirical findings and, finally, Sect. 5 concludes and discusses the strengths and limitations of this study.

## Methodology

### Duration model

We estimate two competing risk regression models in different contexts. Model 1 examines the school-to-work transition of graduates from vocational and higher education. Model 2 focuses on individuals who secured initial employment following graduation and evaluates the subsequent labor market dynamics. Analyzing the school-to-work transition requires tracking individuals’ socio-economic situations over time, despite uncertainty about when changes will occur. Censoring poses a challenge when transitions are not observed for all individuals within the time window covered by our data. Since some individuals may still transition into one of the observed outcomes later, employment rates derived from an arbitrary time window may not capture all relevant dynamics and could lead to biased inferences.

Survival analysis estimates the probability that an event of interest, such as employment upon graduation, has not yet occurred at a given point in time, addressing the issue of censoring. However, this study considers six competing socio-economic events that an individual may experience instead of employment: self-employment, receiving unemployment insurance, receiving social benefits, receiving illness/disability insurance, pursuing further education, other non-income socio-economic events. Unlike censoring, competing risks permanently alter an individual’s likelihood of experiencing the event of interest. A model that ignores these competing risks would, for example, classify someone who pursues further education as still being at risk for employment, even though their career path has fundamentally changed. Similarly, someone receiving illness/disability benefits may not actively be seeking employment, making them no longer part of the true at-risk population.

Ignoring these competing risks can lead to biased probability estimates. In the short term, it may underestimate the probability of employment as the at-risk population can include individuals who are no longer truly at risk, for example, because these individuals have already transitioned into self-employment. Over time, this misclassification can overestimate the probability of employment because the cumulative incidence function continues to assign probabilities to individuals who were never truly at risk. As a result, errors accumulate in long-term cumulative incidence estimates, misrepresenting the likelihood of employment after graduation.

The hazard of experiencing any of the seven considered events at time *t* can be expressed as:

$$\:\mathrm{H}\left(\mathrm{t}\right)=\:{\mathrm{h}}_{1}\left(\mathrm{t}\right)+{\mathrm{h}}_{2}\left(\mathrm{t}\right)+\:\cdots\:+\:{\mathrm{h}}_{7}\left(\mathrm{t}\right)$$

where $\:{\mathrm{h}}_{s}\left(t\right)$ represents the hazard for event s. The probability of observing event *s* at time *T* is given by:

$$\:P\left({\mathrm{h}}_{s}\right)=\frac{{\mathrm{h}}_{s}\left(\mathrm{T}\right)}{{\mathrm{h}}_{1}\left(\mathrm{T}\right)+{\mathrm{h}}_{2}\left(\mathrm{t}\right)+\cdots\:+\:\:{\mathrm{h}}_{s}\left(\mathrm{T}\right)}$$

Conventional survival analyses focuses on the survival function for event *s*, which is:

$$\:P\left(T>t,\:S=s\right)$$

This represents the probability that the event of interest has not yet occurred by time *t*. However, when competing risks are present, it is more appropriate to use a cumulative incidence function (*CIF*), as it represents the probability that the time-to-event is less than or equal to time *t* for each competing risk:

$$\:{CIF}_{s}\left(t\right)=\:P\left(T\le\:t,s=1\right)$$

Fine, Gray [19] introduced a model that estimates cumulative incidence functions while accounting for the presence of competing risks. Their model produces unbiased estimators and allows for the inclusion of covariates to quantify their effects on event probabilities. The sub-hazard function for event *s* is given by:

$$\:{h}_{s}^{*}\left(t\right)=\underset{\delta\:\to\:0}{\mathrm{lim}}\frac{P\left(t<T\le\:t+\delta\:,S=s\:|\:T>t\:\cup\:(T\le\:t\cap\:S\ne\:s)\right)}{\delta\:}$$

Maintaining the idea that the time-to-event is less than or equal to *t*, this model measures the sub-hazard function $\:{h}_{s}^{*}\left(t\right)$. This allows for the calculation of the *CIF* using the cumulative sub-hazard function $\:{H}_{s}^{*}\left(t\right)$:

$$\:{H}_{s}^{*}\left(t\right)={\int\:}_{0}^{t}{h}_{s}^{*}\left(t\right)dt$$

$$\:{CIF}_{s}\left(t\right)=1-{e}^{-{H}_{s}^{*}\left(t\right)}$$

A semiparametric regression is used to quantify the effects of covariates on the sub-hazard using the following regression equation below:

$$\:\:{h}_{s}^{*}\left(t|x\right)={\:{h}_{s}^{*}\left(t\right)}_{0}\bullet\:{e}^{x{\prime\:}\beta\:}$$

These competing risks duration regression models are estimated independently for each outcome because the sub-hazard for each event *s* is modeled separately in the Fine-Gray framework, ensuring that each $\:{CIF}_{s}\:$is correctly estimated relative to its own event of interest while accounting for competing risks. Since each sub-hazard function $\:{h}_{s}^{*}\left(t\right)$ contributes independently to the likelihood, we can conveniently estimate these functions separately as it does not alter the estimated coefficients or standard errors. The variance-covariance matrix for each event type is derived solely from the information matrix of its own likelihood function, meaning that simultaneous estimation would not provide additional efficiency gains. Furthermore, because the competing risks framework already ensures that individuals experiencing competing events remain in the risk set with adjusted weighting, separate estimation maintains the correct risk structure without loss of information.

### Matching procedure

Since differences in age, gender, educational and migration background are likely correlated with both having VI and the considered SEOs, a naive comparison of the declarants with VI against (a randomly drawn sample of) the Dutch population would generate results that are at least partly driven by differences in these underlying characteristics. The objective is to evaluate the differences in SEOs independently of factors like educational level, to determine if there are unique differences in SEOs attributable to VI alone. To address these underlying differences, we calculate propensity scores based on key observable characteristics to create a 1:1 nearest neighbor matched control group.^1^

In the school-to-work transition competing risk model the matching variables include gender, year of graduation, migrant status, education level, and standardized measures of academic achievement. For the post-employment entry competing risk model, we repeat the matching process conditional on employment using the same approach as in the first model, with the year of graduation replaced by the year of initial employment.^2^

We did not include the potentially endogenous variable age as a matching variable. If declarants with VI graduate later, then this can be considered an outcome and the matching on age might not realize the equivalence desired. We do include age upon graduation as a control variable to examine how this changes the estimation results, because age upon graduation can reflect important, but unobserved mechanisms of ability (e.g., higher age due to repeating grades for the matched control group) or effort (e.g., higher age reflecting barriers in education for declarants with VI) that could play out differently for both groups.

Importantly, we assume that declarants with VI and their matched counterparts exhibit the same time-dependency. To empirically verify this assumption, we estimate the competing risks regression models separately for both groups and test whether the time parameters differ statistically and significantly between them.

## Data & descriptive statistics

### Data

For the analyses individual-level administrative register data from Statistics Netherlands (SN) is used. These data combine registers from a variety of governmental and non-governmental sources, including municipal registration data and registration data related to graduation, welfare, employment, healthcare, and insurance. Our final dataset is the result of linking 11 individual datasets. Each includes a unique personal identifier that allows linkage across datasets at the individual level. Because this study focuses on the school-to-work transition, only cases with a graduation date from vocational or higher education were selected. Due to data availability, this covers the period between 1999 and 2019.

To identify the subpopulation of people with VI who claimed reimbursement for sensory disability care in the Netherlands (declarants with VI), health insurance records from 2015 to 2019 were used. Health insurance is mandatory in the Netherlands.^3^ The eligibility for this type of care is based on the definition of VI by the Netherlands Ophthalmological Society. According to these criteria, a VI is present if (1) visual acuity in the best eye is below 30%, (2) the visual field spans below 30 degrees, or (3) the visual acuity in the best eye is between 30 and 50%, but with severe limitations in daily functioning [20]. Sensory disability care for people with VI includes interventions aimed at optimizing independent functioning (rehabilitation) and learning to cope with the disability. It does not include medical care, psychiatric care, or complex long-term residential care [21].

#### Graduation information

We focus on the population of graduates to isolate the school-to-work transition. In the Netherlands, school attendance is compulsory for all children aged 5 to 16. Additionally, young people aged 16 to 18 must obtain a basic qualification before leaving school. According to the International Standard Classification of Education (ISCED) framework, satisfying this basic qualification requirement in the Dutch education system corresponds to graduating from a program at ISCED level 3 or higher.

While academic secondary education programs (in Dutch: HAVO and VWO) are at ISCED level 3 and meet the basic qualification requirement, the vast majority of these graduates do not transition directly to the labor market but instead pursue further education.^4^ Therefore, our analyses include individuals who graduated from vocational education (ISCED 2/3) or higher education (ISCED 5/6/7). It follows that the categorical educational variable used in our empirical models is defined as: low (ISCED-2, in Dutch: MBO 1), middle (ISCED-3, in Dutch: MBO 2–4), and high (ISCED 5–7, in Dutch: HBO/WO bachelor/master).

We select cases where we can establish a graduation date from vocational or higher education between 1999 and 2019. Our analysis is thus based on this subpopulation of declarants with VI who graduated from vocational or higher education, identifying a total of 2,412 individuals.

To construct a matched control group, we extract a random sample of 2 million people from the Dutch general population. Again, we selected only those for whom we could establish a graduation date from vocational or higher education between 1999 and 2019. This resulting sample of 309,176 individuals formed the basis for constructing a credible control group via a nearest neighbor matching procedure.

#### Outcome variables and background characteristics

In the longitudinal constructed dataset, we observe age, gender, migrant status, highest achieved educational level, and high-school GPA as a summary measure of previous academic achievement. Migrant status is defined following the definition of SN. Individuals whose parents were both born in the Netherlands are defined as non-migrants. First-generation immigrants are those who were born abroad with at least one non-Dutch parent, while second-generation immigrants are those born in the Netherlands with at least one parent born abroad.

The main outcome variable of interest is the socio-economic category in a given month (SECM), as defined by SN. The SECM variable is defined based on the primary source of income, or time allocation in the absence of income, and is derived from a dummy (yes/no) coding of 13 distinct SEO categories related to whether an individual participated in paid labor, received (social or insurance) benefits, and/or engaged in formal schooling. We reorganize these SEOs into the following categories: (1) employment, (2) self-employment, (3) unemployment insurance, (4) social benefits, (5) illness/disability insurance, (6) attending further education, and (7) other without income. Table 1 provides definitions for each SEO category.

**Table 1:** Definitions of socio-economic outcomes

SEO	Definition
Employment	Employed by an employer other than themselves
Self-employment	Entrepreneurs and other self-employed e.g., freelancers
Unemployment insurance	Eligible when unemployed for as many months as one previously worked in years
Social benefits	Social assistance benefits and other social benefits (including Wajong, support for older and partially disabled people, assistance for artists and self-employed, war and resistance pensions)
Illness/disability insurance	Illness and disability insurance including premature pension benefits
Further education	Full-time education, only marginal additional earnings
Other without income^a^	Neither in employment nor in education or training; no income of their own. When person dropped out of any of the recorded SEOs

^a^outcome only present in second Model - after employment entry

The data also allow to investigate potential secondary sources of income. This is represented by the aforementioned dummy-coded variables on all potential sources of income. When a source of income is coded affirmatively, but not as the dominant source of income, it is labelled a secondary source of income. The results on secondary sources of income are presented and discussed in Sect. 5.3.

### Descriptive statistics

Table 2 presents the pre-matching descriptive statistics for both the 2,412 graduated declarants with VI and the 309,176 random sample of graduates drawn from the Dutch general population. These differences reveal that graduated declarants with VI are, on average, older at graduation, consist of a higher proportion of females, more frequently graduate from vocational degree programs, and are less likely to have a first-generation immigrant background. These differences emphasize the importance of applying the aforementioned matching procedure and covariate control methodologies when comparing the two groups in terms of their SEOs.

**Table 2:** Post graduation descriptive statistics before matching

	General population sample (*n* = 309,176)	Declarants with VI (*n* = 2.412)	Combined	*P*-value	
Year of birth	1985.70	1981.10	1985.66	0.0000	***
Year of graduation	2012.03	2011.28	2012.02	0.0000	***
Mean age at graduation (years)	26.50	30.18	26.53	0.0000	***
Gender (%)					
*Female*	52.75%	56.05%	52.78%	0.0012	***
*Male*	47.25%	43.95%	47.22%	0.0012	***
Level of education (%)					
*Low or no info*	6.88%	9.37%	6.89%	0.0000	***
*Middle*	44.61%	49.67%	44.65%	0.0000	***
*High*	48.51%	40.96%	48.46%	0.0000	***
GPA (mean. 1–10)	6.53	6.51	6.53	0.60	
Migration background (%)					
*Netherlands*	76.04%	77.40%	76.05%	0.1106	
*First generation*	11.25%	9.25%	11.23%	0.0007	***
*Second generation*	12.71%	13.35%	12.72%	0.3596	
Urbanicity ^a^	2.332	2.397	2.333	0.0125	*

^a^denotes a matching quality control variable that was not included in the matching procedure

*p < 0.05.** *p* < 0.01. *** *p* < 0.001

## Results

### Findings on the school-to-work transition after graduation

#### Nearest-Neighbor matching

Table 3 presents descriptive statistics for graduated declarants with VI and the matched control group. After matching, both groups consist of 2,412 individuals each, and there are no significant mean group except for age. As explained in Sect. 2.1, age was an endogenous matching variable, and therefore we examine how the estimation results of the duration models, and particularly the estimated coefficient for the indicator variable denoting whether a person has a VI, change with the inclusion of age.

**Table 3:** Post graduation descriptive statistics after matching

	Matched sample (*n* = 2.412)	Declarants with VI (*n* = 2.412)	Combined	*P*-value	
Year of birth	1983.86	1981.10	1982.48	0.0000	***
Year of graduation	2011.28	2011.28	2011.28	0.9767	
Mean age at graduation (years)	27.42	30.18	28.80	0.0000	***
Gender (%)					
*Female*	56.05%	56.05%	56.05%	1.000	
*Male*	43.95%	43.95%	43.95%	1.000	
Level of education (%)					
*Low or no info*	9.37%	9.37%	9.37%	1.000	
*Middle*	49.67%	49.67%	49.67%	1.000	
*High*	41.00%	41.00%	41.00%	1.000	
GPA (mean. 1–10)	6.51	6.51	6.51	0.9766	
Migration background (%)					
*Netherlands*	77.40%	77.40%	77.40%	1.0000	
*First generation*	9.25%	9.25%	9.25%	1.0000	
*Second generation*	13.35%	13.35%	13.35%	1.0000	
Urbanicity^a^	2.368	2.397	2.382	0.4228	

^a^denotes a matching quality control variable that was not included in the matching procedure

*p < 0.05. ** *p* < 0.01. *** *p* < 0.001

#### Socio-Economic outcomes (SEOs)

Before presenting the estimation results of the duration model simultaneously for the various SEOs we first compare the differences in SEO proportions between the declarants with VI and the matched control group.

Table 4 presents the frequencies of each SEO for both groups after the first school-to-work transition, along with the mean cumulative incidences. The declarants with VI have lower cross-sectional employment rates compared to the matched control group, with no significant difference in self-employment rates. Generally, the declarants with VI appear more reliant on governmental support, primarily through illness/disability insurance and social benefits, though there is no significant difference in regular unemployment insurance. The proportion of individuals attending further education upon graduation is similar across both groups. Additionally, a smaller proportion of declarants with VI does not transition into any of these categories compared to the matched control group, meaning they are neither in education or labor nor eligible for insurance or benefits but remain after graduation in a other, no income status.

**Table 4:** Prevalence of economic outcome per group and mean cumulative incidence post graduation

	Matched *n*, (%) (*N* = 2.412)	Declarants with VI n, (%) (*N* = 2.412)		X^2^			*p*-value	
	Mean cumulative incidence		Mean cumulative incidence		Mean difference	t		
Socio-economic category								
No change	271 (11.24%)		174 (7.22%)	21.14			0.000	***
	-		-					
Employment	1571 (65.13%)		1250 (51.87%)	36.53			0.000	***
	0.533		0.419		−0.114	11.164	0.000	***
Self-employment	81 (3.36%)		68 (2.82%)	1.13			0.287	
	0.018		0.015		−0.003	1.59	0.114	
Unemployment insurance	28 (1.16%)		25 (1.04%)	0.17			0.287	
	0.006		0.006		0	1.182	0.240	
Social benefits	67 (2.78%)		418 (17.34%)	254.02			0.000	***
	0.016		0.121		0.105	−35.592	0.000	***
Illness/disability insurance	32 (1.32%)		104 (4.32%)	38.12			0.000	***
	0.007		0.026		0.019	−11.345	0.000	***
Attending education	362 (15.00%)		371 (15.39%)	0.11			0.740	
	0.099		0.103		0.004	−1.269	0.205	
	*100.00%*		*100.00%*					

*p < 0.05. ** *p* < 0.01. *** *p* < 0.001

#### Estimation results of the competing risks duration model

As indicated, a simple comparison of the differences in SEOs between both groups may be misleading due to censoring. Additionally, in the analysis presented here, we further control for the potentially confounding effect of age at graduation in the competing risks regression analysis. Conducting the analysis without inclusion of the age covariates did not result in significant changes to the estimated effects nor our conclusions with regards to the effect of VI. The resulting estimates omitting the age covariates can be found in the supplement Table s2. Table 5 shows the relevant effect sizes from the competing risks regression analysis upon graduation.

**Table 5:** Competing risks regression estimates for the school-to-work transition

Economic outcome	(1) Employment		(2) Self-employment		(3) Unemployment insurance		(4) Social benefits		(5) Illness and disability insurance		(6) Education	
	B (SE)	p	B (SE)	p	B (SE)	p	B (SE)	P	B (SE)	p	B (SE)	p
*Visual impairment*	−0.406 (0.038)	0.000 ***	−0.359 (0.168)	0.033 *	−0.176 (0.280)	0.530	2.028 (0.133)	0.000 ***	0.593 (0.205)	0.004 **	0.134 (0.073)	0.068
Age at graduation	0.183 (0.012)	0.000 ***	0.082 (0.017)	0.000 ***	−0.053 (0.032)	0.490	0.346 (0.055)	0.000 ***	0.096 (0.027)	0.000 ***	−0.113 (0.017)	0.000
Higher order polynomials of age	Included up to 2nd order		Not included		Not included		Included up to second order		Not included		Not included	
Gender – woman	−0.025 (0.038)	0.520	0.244 (0.169)	0.150	0.197 (0.288)	0.490	−0.011 (0.092)	0.910	−0.100 (0.180)	0.580	0.000 (0.075)	1.000
Migration – first generation	−0.460 (0.073)	0.000 ***	−0.413 (0.332)	0.210	0.669 (0.396)	0.091	0.318 (0.140)	0.023 **	−0.219 (0.372)	0.560	0.536 (0.136)	0.000 ***
Migration – second generation	−0.442 (0.073)	0.000 ***	0.059 (0.255)	0.820	0.423 (0.393)	0.280	0.056 (0.128)	0.660	0.280 (0.271)	0.300	0.489 (0.136)	0.000 ***
Education – middle	0.280 (0.075)	0.002 ***	1.548 (0.583)	0.008 ***	1.184 (1.034)	0.250	−0.756 (0.144)	0.000 ***	0.642 (0.406)	0.110	0.331 (0.124)	0.007 **
Education - high	0.030 (0.075)	0.69	1.756 (0.591)	0.003 ***	1.942 (1.037)	0.061	−1.009 (0.153)	0.000 ***	0.728 (0.405)	0.072	−0.235 (0.156)	0.130
GPA	0.030 (0.033)	0.470	Not inc		Not inc		0.033 (0.093)	0.720	Not inc		Not inc	
Birthyear	−0.007 (0.005)	0.18	0.032 (0.016)	0.050	−0.071 (0.027)	0.009 **	0.030 (0.110)	0.007	−0.029 (0.026)	0.260	0.051 (0.009)	0.000 ***
*N*	4822		4822		4822		4822		4822		4822	

*p < 0.05. ** *p* < 0.01. *** *p* < 0.001

Consistent with earlier observations, we find a negative association between having VI and employment. Additionally, the association with self-employment reaches significance. The most substantial group difference was observed in social benefits, where there is a positive association between having VI and the likelihood of receiving these benefits. Also the reception of illness and disability insurance was significantly more likely for declarants with VI. The duration model does not point to differences between declarants with VI and the matched control group in unemployment insurance or continuing further education upon graduation.

Returning to Table 4, we also find the mean cumulative incidence function for each group and may compare these to the cumulative incidence estimated at day 365 after graduation reported in Table 6. By estimating the proportion of individuals who have experienced the event of interest at time t, the CIF can be interpreted as probability of experiencing the event by time of estimation. According to these estimates and averaged across days after graduation, 53.3% of the matched group finds their way into employment compared to only 42.9% of declarants with VI, resulting in a significant group difference of 11.4%. This corresponds to 64.1% and 51% at the 365 days point after graduation, respectively. The group difference in mean cumulative incidence for receiving social benefits is, with 10.5%, reflecting the difference in employment but in the opposite direction. 12.1% of declarants with VI and only 1.6% of the matched sample according to these estimates. On day 365 post-graduation the group difference is even larger with 17.34% of declarants with VI and 2.8% of the matched sample. According to the mean cumulative incidence function, we find 0.7% of the matched sample as recipients of illness/disability insurance, more than three times this, 2.6% was estimated for the declarants with VI. This share increases among both groups as evidenced on day 365 after graduation with 1.3% for the matched sample and 4.3% for declarants with VI.

**Table 6:** Cumulative incidence 365 days post graduation

Socio-Economic Outcome	Matched sample	Declarants with VI
Employment	0.6414	0.5095
Self-employment	0.0336	0.0282
Unemployment insurance	0.0116	0.0104
Social benefits	0.0278	0.1734
Illness/disability insurance	0.0133	0.0432
Attending education	0.1501	0.1539

**Fig. 1:**
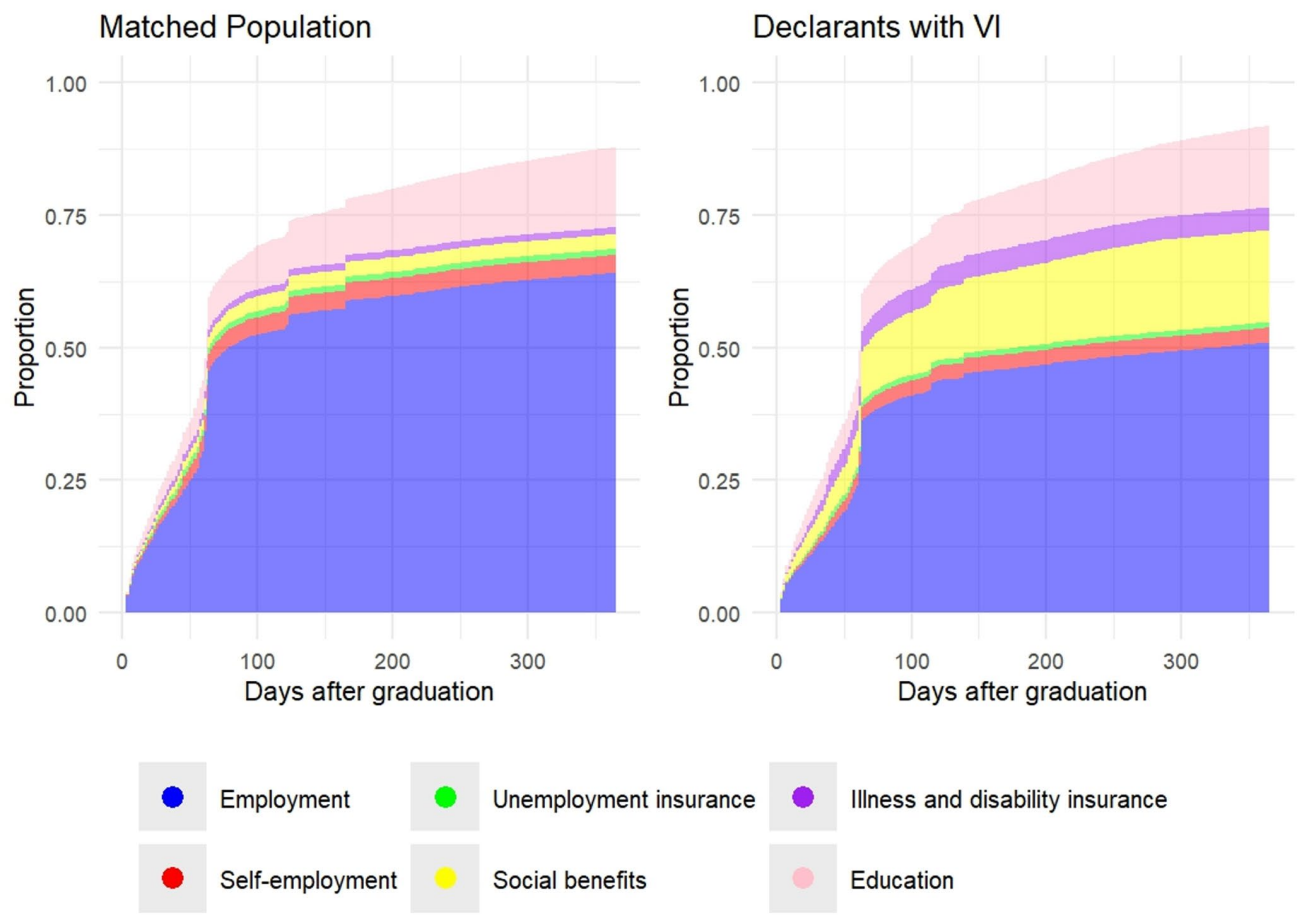
Transitions into SEOs across the first year after graduation

Given a maximum 14.5% point difference in receipt of social benefits and an estimated mean cost of approximately 18 thousand euros per recipient per year,^5^ our limited sample of claimants (*n* = 2412) with visual impairments who we observed in their school-to-work transition phase already corresponds to an additional 6 million euros in annual social benefit expenditures. Reducing this gap across the broader labor force population would thus not only significantly lower the government’s welfare burden but also increase wage tax revenues. These differences are therefore highly relevant for social and economic policy. Comparing the estimates displayed in Table 5 with those in Table s3 in the supplementary material suggests that the time dynamics regarding SEO transitions are similar across groups. This similarity is also reflected in Fig. 1, which illustrates the effect of having VI on the transition to various SEOs throughout the first year after graduation.^6^

### Findings on labor market dynamics after successful initial employment after graduation

#### Nearest-Neighbor matching

Tables 7 and 8 show the pre- and post-matching results, respectively, given successful initial employment after graduation. The declarants with VI that successfully transitioned into paid labor after graduation consists of 1,252 individuals (51.9%), while for the random sample of the Dutch population, this amounts to 202,126 persons (65.4%).

**Table 7:** Post employment entry descriptive statistics before matching

	Matched sample (*n* = 202,126)	Declarants with VI (*n* = 1,252)	Combined	*p*-value	
Year of birth	1984.81	1978.36	1984.77	0.0000	****
Year of first job	2012.90	2012.80	2012.90	0.0000	****
Mean age at first job (years)	27.04	32.42	27.07	0.0000	****
Gender (%)					
*Female*	53.15%	56.95%	53.18%	0.0070	***
*Male*	46.84%	43.05%	46.82%	0.0070	**
Level of education (%)					
*Low or no info*	4.00%	9.50%	4.03%	0.0000	****
*Middle*	45.44%	46.09%	45.44%	0.6461	
*High*	50.56%	44.41%	50.52%	0.0001	***
GPA (mean. 1–10)	6.53	6.52	6.53	0.885	
Migration background (%)					
*Netherlands*	81.68%	81.79%	81.68%	0.9215	
*First generation*	7.49%	8.15%	7.50%	0.3986	
*Second generation*	10.83%	10.06%	10.82%	0.3715	
Urbanicity^a^	2.442	2.465	2.442	0.518	

^a^denotes a matching quality control variable that was not included in the matching procedure

*p < 0.05. ** *p* < 0.01. *** *p* < 0.001

**Table 8:** Post employment entry descriptive statistics after matching

	Matched sample (*n* = 1,252)	Declarants with VI (*n* = 1,252)	Combined	*p*-value	
Year of birth	1982.92	1978.36	1980.64	0.0000	****
Year of first job	2010.79	2010.78	2010.79	0.980	
Mean age at first job (years)	27.86	32.42	30.14	0.0000	****
Gender (%)					
*Female*	57.03%	56.95%	56.99%	0.9678	
*Male*	42.97%	43.05%	43.02%	0.9678	
Level of education (%)					
*Low or no info*	9.50%	9.50%	9.50%	1.0000	
*Middle*	46.09%	46.09%	46.09%	1.0000	
*High*	44.41%	44.41%	44.41%	1.0000	
GPA (mean. 1–10)	6.57	6.52	6.54	0.6227	
Migration background (%)					
*Netherlands*	81.71%	81.79%	81.75%	0.9588	
*First generation*	8.23%	8.15%	8.19%	0.9419	
*Second generation*	10.06%	10.06%	10.06%	1.0000	
Urbanicity^a^	2.457	2.465	2.461	0.863	

^a^denotes a matching quality control variable that was not included in the matching procedure

* *p* < 0.05. ** *p* < 0.01. *** *p* < 0.001

The differences in baseline characteristics appear to be similar to those found in the initial Table 3 sample. After applying a nearest neighbor matching approach, both groups consist of 1,252 people with no significant mean differences between the declarants with VI and the matched control group, given successful initial employment after graduation. The exception is again age, which was not included in the matching procedure because of its potentially endogenous character (see Sect. 4.1.1).

#### Socio-Economic outcomes

Table 9 shows the distribution of SEOs for individuals after securing initial employment. We observe that a smaller proportion of declarants with VI transition into different SEOs compared to the matched control group. However, a larger share of the declarants with VI experiences a change in employment or acquires a secondary source of income while maintaining employment as their primary source of income (i.e., no change in SEO). There is, however, a relative overrepresentation of the declarants with VI receiving social benefits and illness or disability insurance compared to the matched control group. Conversely, we observe an underrepresentation of declarants with VI in further education, as well as in those who are neither employed, pursuing education, nor receiving social or insurance benefits compared to the matched control group.

**Table 9:** Prevalence of economic outcome per group and mean cumulative incidence post employment entry

	Matched *n*, (%) (*N* = 1252)	Declarants with VI *n*, (%) (*N* = 1252)	X^2^			*p*-value	
	Mean cumulative incidence	Mean cumulative incidence		Mean difference	t		
Socio-economic category							
No change	498 (39.78%)	295 (23.56%)	51.97			0.000	***
	-	-					
Employment	370 (29.55%)	531 (42.41%)	28.77			0.000	***
	0.335	0.359		0.024	−2.664	0.007	**
Self-employment	26 (2.08%)	17 (1.36%)	1.88			0.169	
	0.018	0.009		-0.009	4.198	0.000	***
Unemployment insurance	79 (6.07%)	82 (6.55%)	0.06			0.813	
	0.058	0.051		−0.007	2.425	0.016	*
Social benefits	11 (0.87%)	52 (4.15%)	26.68			0.000	***
	0.008	0.032		0.024	−11.865	0.000	***
Illness/disability insurance	24 (2.16%)	94 (7.51%)	41.53			0.000	***
	0.019	0.056		0.037	−14.062	0.000	***
Attending education	57 (4.47%)	37 (2.96%)	4.26			0.039	*
	0.051	0.026		−0.025	6.577	0.000	***
Other without income	187 (14.78%)	144 (11.50%)	5.59			0.018	*
	0.158	0.097		−0.061	11.581	0.000	***
	*100.00%*	*100.00%*					

*p < 0.05. ** *p* < 0.01. *** *p* < 0.001

#### Duration model

The competing risks regression model post employment entry reveals that once initial employment was secured, and given a change in employment status occurred, individuals with VI exhibit a greater likelihood of maintaining employment as primary source of income. Nonetheless, this analysis revealed no differences for transitioning into unemployment insurance, pursuing further education, the other no income category, or self-employment between declarants with VI and the matched control group. However, having VI is positively associated with receiving income through social benefits and illness or disability insurance. The corresponding effect sizes are detailed in Table 10. The estimation results without the inclusion of age covariates led to the same conclusions. These results are provided in the supplementary material, Table s4.

**Table 10:** Competing risks regression estimates post employment entry

Economic outcome	(1) Employment		(2) Self-employment		(3) Unemployment insurance		(4) Social benefits		(5) Illness and disability insurance		(6) education		(7) Other no income	
	B (SE)	p	B (SE)	p	B (SE)	p	B (SE)	P	B (SE)	p	B (SE)	p	B (SE)	p
*Visual impairment*	0.409 (0.069)	0.000 ***	−0.613 (0.333)	0.065	0.092 (0.164)	0.580	1.662 (0.334)	0.000 ****	1.130 (0.245)	0.000 ***	−0.156 (0.217)	0.470	−0.043 (0.113)	0.700
Age at Job entrance	−0.042 (0.025)	0.099	0.224 (0.124)	0.071	0.219 (0.338)	0.520	0.288 (0.379)	0.440 ***	0.104 (0.297)	0.780	−0.609 (0.434)	0.160	0.026 (0.197)	0.900
Higher order polynomials of age	Included up to 2nd order		Included up to 2nd order		Included up to 3rd order		Included up to 3rd order		Included up to 3rd order		Included up to 3rd order		Included up to 3rd order	
Gender – woman	0.160 (0.068)	0.018 *	0.331 (0.332)	0.320	−0.367 (0.161)	0.023 *	−0.607 (0.263)	0.021 *	0.488 (0.200)	0.014 *	−0.269 (0.223)	0.230	−0.035 (0.115)	0.760
Migration – first generation	−0.050 (0.123)	0.690	−0.606 (0.733)	0.410	0.367 (0.248)	0.140	−0.239 (0.468)	0.610	−0.603 (0.335)	0.860	1.030 (0.342)	0.019	0.495 (0.195)	0.400
Migration – second generation	−0.012 (0.118)	0.920	0.420 (0.445)	0.350	−0.130 (0.282)	0.640	−0.517 (0.521)	0.320	0.334 (0.312)	0.280	0.182 (0.330)	0.580	0.146 (0.175)	0.250
Education – middle	−0.267 (0.105)	0.011 *	Not inc		0.436 (0.335)	0.190	−0.374 (0.386)	0.330	0.249 (0.324)	0.440	0.035 (0.342)	0.920	−0.522 (0.206)	0.011 *
Education - high	−0.250 (0.106)	0.018 *	0.897 (0.338)	0.008 **	0.314 (0.328)	0.340	−0.766 (0.409)	0.061	0.180 (0.346)	0.600	−0.254 (0.441)	0.570	0.163 (0.212)	0.440
GPA	−0.058 (0.078)	0.460	0.159 (0.303)	0.600	−0.244 (0.110)	0.027 *	0.184 (0.324)	0.570	0.302 (0.449)	0.500	0.093 (0.109)	0.400	−0.107 (0.117)	0.360
Birthyear	−0.044 (0.008)	0.000 ***	−0.035 (0.029)	0.230	−0.057 (0.020)	0.006 **	−0.028 (0.028)	0.310	0.008 (0.024)	0.75	−0.004 (0.030)	0.910	−0.062 (0.014)	0.000 ***
*N*	2498		2498		2498		2498		2498		2498		2498	

* *p* < 0.05. ** *p* < 0.01. *** *p* < 0.001

Returning to Table 8 and comparing with Table 11 for the mean cumulative incidences and cumulative incidence at 365 days after employment entry, we see 33.5% of the matched general population share and 35.9% of declarants with VI maintaining employment as dominant source of income. This group difference widened 365 days after employment entry this to 29.6% and 38.1%, respectively. Averaged across days after employment entry, 0.8% of the matched sample shifted into social benefits, among declarants with VI this figure is 3.2%, which increased substantially to 17.3% at the 365 days mark, while remaining around 0.9% among the matched sample. According to the mean cumulative incidence as well as at 365 days after graduation, 1.9% of the matched sample are recipients of illness/disability insurance. For declarants with VI this amounts to 5.6% averaged across days after employment entry and kept increasing to 7.5% on the 365 day mark. Lastly, the mean cumulative incidence of the matched leave employment without having another source of income was 15.8%. This figure amounts to 9.7% among declarants with VI. On day 365 after employment entry, this gap narrowed and we observe this status among 14.9% of the matched sample and 11.5% of declarants with VI.

**Table 11:** Cumulative incidence 365 days post employment entry

Socio-Economic Outcome	Matched sample	Declarants wit VI
Employment	0.2955	0.3810
Self-employment	0.0208	0.0136
Unemployment insurance	0.0631	0.0655
Social benefits	0.0088	0.1734
Illness/disability insurance	0.0192	0.0751
Attending education	0.0455	0.0296
Other no income	0.1494	0.1150

In addition to the substantial difference in social benefits observed in the post-graduation analysis, the post-employment entry results show a maximum difference of 5.6% points in disability insurance receipt. With an estimated mean cost per recipient of 14 thousand euros per year,^6^ this difference leads to an additional annual cost of 1 million euros for the Dutch government for already only our limited sample of 1,252 individuals with visual impairments.

**Fig. 2:**
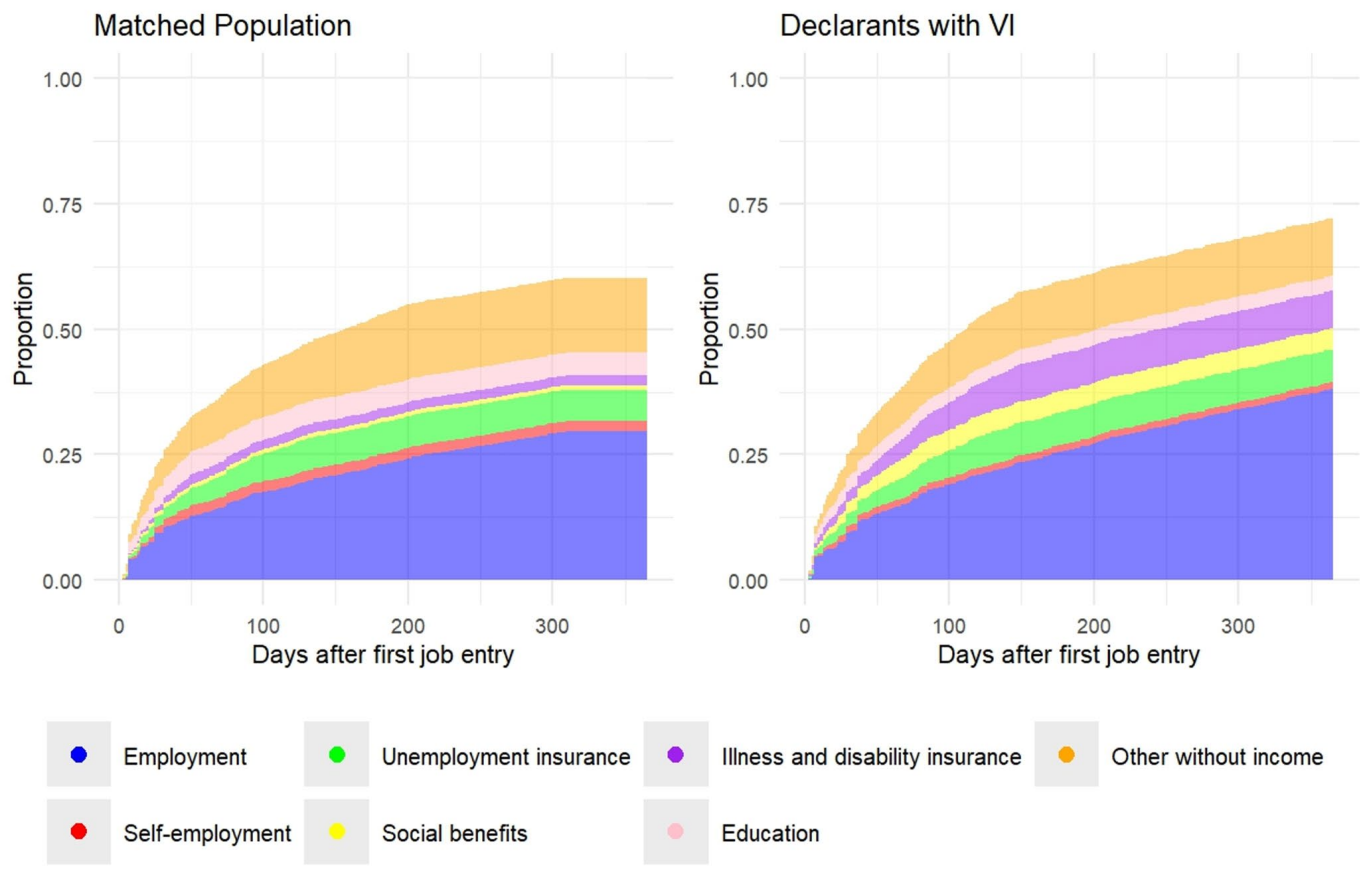
Transitions into SEOs across the first year after initial employment

Figure 2 and S2 show the distribution of Social and Economic Outcomes (SEOs) for the declarants with VI and the matched control group, analyzed separately over the first 365 and 750 days following initial employment. A preliminary visual analysis of these figures suggests potentially different time dynamics between the two groups, with somewhat slower transitions observed for the declarants with VI. As do the differences in mean cumulative incidence function compared to those at the 365 days mark. However, the effect size estimates of the independent variables remain the same when estimating the censoring distribution of each group separately. The respective estimates are presented in Table s5.

### Secondary sources of income

**Fig. 3:**
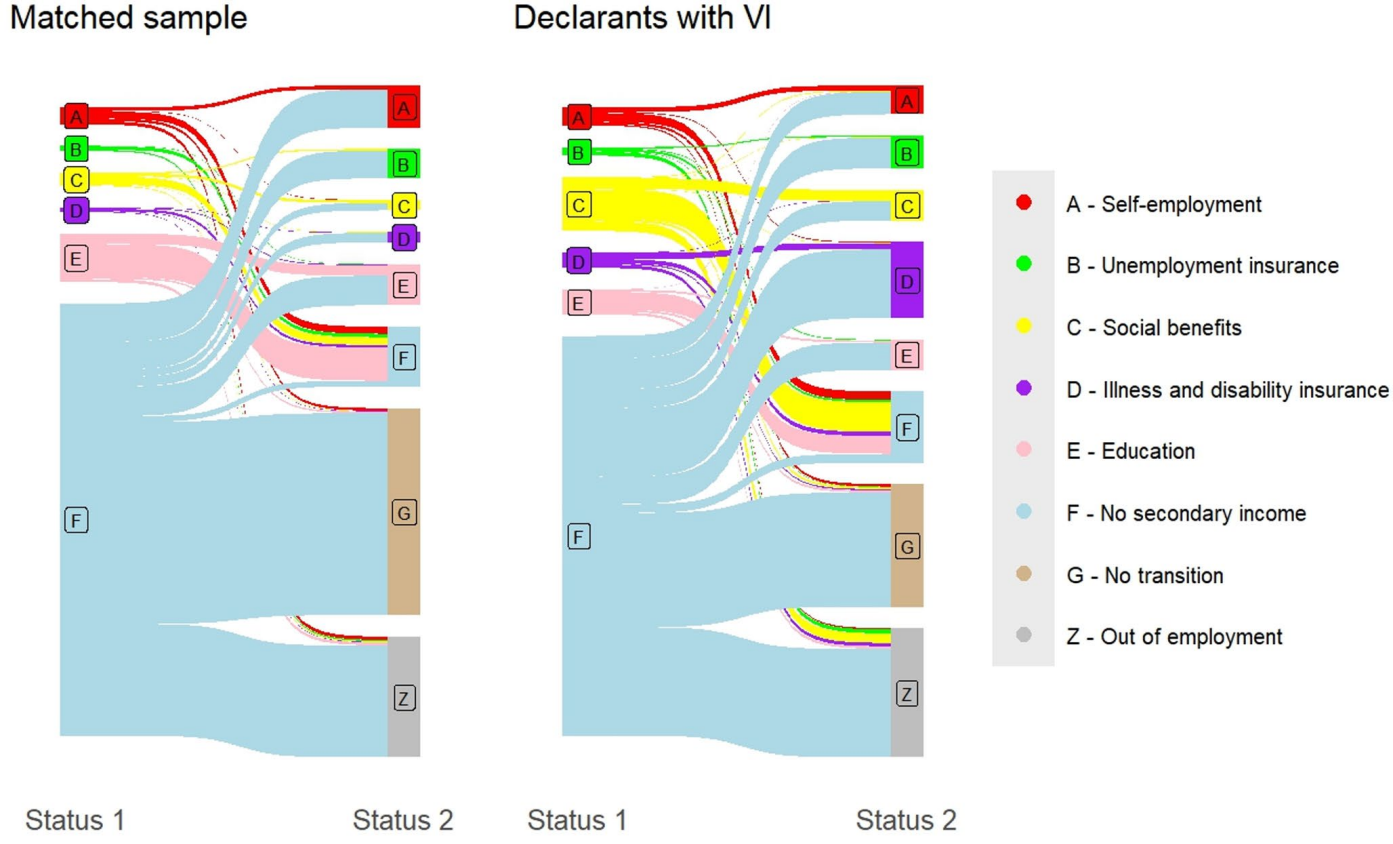
Dynamics in secondary incomes next to employment per group

Focusing on the distributions of potential secondary sources of income means that these analyses are conducted for graduates whose primary income is derived from employment. Figure 3 shows the dynamics in secondary sources of income between the initial and subsequent states (i.e., status 1 and status 2) observed after graduation.^7^ Based on this plot, we can conclude that the declarants with VI are more likely to obtain income through secondary sources compared to the matched control group.

Post-graduation (i.e., status 1), the most significant group differences are found for social benefits, where the declarants with VI are overrepresented (*t*(1879.6) = −8.0002, *p* < 0.01) compared to the matched control group. The declarants with VI are also overrepresented with respect to secondary income from illness and disability insurance (*t*(1852.5) = −4.2997, *p* < 0.01) compared to the matched control group. In contrast, the declarants with VI with employment are less likely to be also attending further education (*t*(1852.5) = 3.1636, *p* < 0.01) compared to the matched control group. For self-employment no group difference was found post-graduation (*t*(2496.7) = −0.44379, *p* = 0.65), nor for unemployment insurance (*t*(2327.2) = −1.679, *p* = 0.09).

When evaluating the secondary income sources for the subsequent state observed post-employment entry (i.e., status 2), we see transitions from one secondary income status to another for 875 declarants with VI (69.9%), versus 697 people (55.7%) form the matched control group. The most significant group difference was the number of people experiencing no changes. While some individuals in both groups exited employment, the declarants with VI were more likely to continue earning at least some income from employment (t(1579.4) = −2.5173, *p* = 0.01) compared to the matched control group.

An increase in secondary income from illness and disability insurances was primarily observed for the declarants with VI (*t*(1502.3) = 11.998, *p* < 0.01). Furthermore, an overrepresentation of the declarants with VI in social benefits secondary incomes remains (t(1656.8) = −5.4371, *p* < 0.01). However, a larger proportion of the matched control group derived some secondary income from self-employment compared to the declarants with VI (*t*(1353.3) = 4.7439, *p* < 0.01). The matched control group also remained overrepresented among those attending further education (*t*(1469.8) = 2.885, *p* < 0.01) compared to the declarants with VI. No difference between both groups was observed regarding secondary income through unemployment insurances (*t*(1575.9) = 0.93324, *p* = 0.35).

## Discussion and conclusion

This study examines the school-to-work transition and post-graduation SEOs for a subpopulation of people with VI, specifically for those who claimed reimbursement for sensory disability care from their health insurances in the Netherlands between 2015 and 2019 (referred to as declarants with VI). It compares their labor market entry patterns with a matched control group. To achieve this, the study utilizes individual-level longitudinal administrative register data, which includes information on SEOs, healthcare reimbursements, educational attainment and performance, and demographic characteristics.

The results show that following graduation, declarants with VI have significantly lower (self-)employment rates compared to the matched control group who are similar on observed characteristics such as gender, year of graduation, migrant status, education level, and standardized measures of academic achievement. The observed group difference corresponds to differential employment rates reported in previous studies [11, 22, 23]. Accordingly, we note a greater dependency on governmental support for declarants with VI. This welfare-dependency was mostly driven by social benefits and sick- and disability- leave, whereas the share of people receiving income through unemployment insurance was similar in both groups.

When limiting the analysis to only those who secured employment as their main source of income, the results highlight that declarants with VI have greater rates of success in maintaining employment compared to the matched control group given a change in employment status. This result emphasizes the importance of a successful school-to-work transition. However, more changes were registered within employment for declarants with VI group. This may indicate that this group may find themselves in greater difficulty retaining their first job after graduation. In addition, declarants with VI experience more dependency on and dynamics in terms of income from secondary sources. This also holds true for those who were able to secure a successful labor market entry after graduation in which employment was the main source of income, as evidenced by larger rates of income received through social benefits and illness and disability insurance.

A first period of employment is considered to be an important prerequisite for transitioning into self-employment [24]. Confirming this, there was no association between VI and transitioning into self-employment following initial employment. Furthermore, we find no association of VI with being in education, nor with the status of receiving no income at all, despite their greater eligibility for social support programs. Either way, this group which is neither in education, employment, or training may be particularly relevant in times of labor shortages as they could be an addition to the labor markets while conventional recruitment and retention seems to have failed them. Eligibility for more beneficial social support programs may also be a reason why we do not see a greater influx into unemployment insurance despite the noted welfare dependence. On the other hand, the share of unemployment insurance recipients is rather small in both groups, thus a difference or absence of such may not be very meaningful. Across both groups, only a minority of graduates will be eligible for unemployment insurance income in the Netherlands since one must have worked for an average of 10 h a week for at least 26 weeks out of the last 36 weeks before becoming unemployed.^8^ Therefore, eligibility is most likely limited to those who followed a part-time degree program.

Time dynamics relating to the labor market entry process do not seem to differ substantially for the declarants with VI considered here. Although they are on average older when entering their first job, this age difference already exists upon graduation. After graduation, the time until a person has transitioned into another SEO does not seem to differ. Accordingly, rather than taking longer to enter into employment upon graduation, declarants with VI takes longer to graduate from school. To some extent this might be because most individuals with VI in our sample will have likely acquired their impairment during their schooling rather than it being a congenital condition [25]. When VI is acquired during school ages, the adjustment process may induce longer educational trajectories.

Nonetheless, post first-employment, the time effects approach to be more gradual than post-graduation. The general time dynamics observed for both groups in our data seem somewhat different from what is reported in previous literature. For example, Baydur, Mukoyama [26] note a median job duration of 14 month in their sample. In our sample, the movements recorded when exiting initial employment have already reached a plateau by approximately 14 months. This, of course, does not reflect the share of employees in our data, who had yet to exit their first employment at time of data analysis. Additionally, as discussed in the limitations, some dynamics from employment, maintaining the dominant source of income as employment may also reflect changes in secondary sources of income, while maintaining the same employment. What might explain the relatively short job duration in our data is the finding by Hyatt, Spletzer [27] that short job durations occur more frequently among younger and less highly-educated workers. Our first job data comes by definition from the younger share of the workforce (i.e., recent graduates) and the declarants with VI displayed lower levels of educational attainment compared to the general population sample before matching.

We also observe more dynamics among the declarants with VI post initial employment, which could reflect greater difficulties in retaining their job. This interpretation is in line with evidence from previous research and affected individuals reporting problems remaining in their current employment [28]. Lastly, our results provide evidence that declarants with VI rely on secondary income sources to a greater extent compared to the matched control group. Meaning that employment as the main source of income, is frequently supplemented by income through social benefits or disability insurances.

Some potential policy implications can be derived from our findings. Evidently, also when observable background characteristics and obtained educational attainment and performance are made comparable, declarants with VI are less likely to secure substantial income through employment. Since their welfare dependency seems to be mostly driven by benefits programs other than regular unemployment insurance, reforms or interventions targeting specifically these programs may be fruitful. Nonetheless, as a systematic review [29] demonstrate, simply restricting the eligibility for specific programs, does not necessarily result in a reduced uptake. Instead, increasing the supply of accessible jobs for the target group may prove fruitful. Furthermore, providing positive incentives and social security for potential failure on the labor market may increase the willingness of individuals with VI to participate in the labor market. Currently, some social welfare programs, such as the WIA [30, 31], penalize recipients who enter the labor market but subsequently lose their employment again. Securing individuals from the costs of potential failure may be a greater incentive to leave benefits programs and enter the labor market than penalizing them for it. Additionally, this research identified a substantial subgroup among both declarants with VI and the matched control group that is neither in employment, education, or training, nor receiving income through additional sources. This group may be particularly relevant for potential recruitment for addressing labor market shortages and their inactivity can indicate specific vulnerability in terms of overall participation in society. Practical implications include that non-profit or non-governmental organizations with the goal of assisting individuals with VI may find it worthwhile to invest more efforts into the school-to-work transition. Frequently, support is offered during education and once problems on labor markets crystallized. Assisting in a smooth school-to-work transition may prevent problems further down the line.

This paper provided specific insights on the labor market prospects of the school-to-work transition of declarants with VI. Expanding the concept of labor market participation beyond a simple employed/unemployed dichotomy allows for more detailed insights relevant for policy-making. The analyses benefited largely from access to individual-level longitudinal administrative register data combined specifically with healthcare data. By exploiting large-scale administrative data, we circumvent several issues and limitations that hamper conventional, small-scale survey studies, such as selective response bias and subjective self-reports. A limitation of our study is it does not include all people with VI in the Netherlands as there may be people with VI who do not declare VI-specific care to their health insurances. However, this sample size in this study is larger than that of any previous research on labor market participation among people with VI, as it encompasses all people form a subpopulation of recent graduates that declared their VI-care to their health insurance. Additionally, our ability to draw causal conclusions about the effect of VI is inherently limited by using matching as identification strategy. The identifying assumption of matching holds that given the observed covariates, there are no systematic differences between individuals with VI and their matched counterparts that may bias the results. While we tested a potentially confounder, urbanicity, pre- and post-matching, without including it in the matching procedure, to see whether other, potentially confounding, variables would also be balanced as a result of the matching process, this approach does not rule out the possibility of unobserved confounders. Another limitation to this study is related to the data available, when individuals remain in a state of employment as the dominant source of income but do experience an income change, we cannot differentiate between whether a change occurred within a job (e.g., working hours), whether a job switch occurred, or whether the change was solely due to changes in secondary income sources. As such, we cannot make clear inferences about to what extent the jobs entered are particularly sustainable. Future research should focus on differential frequencies of potential job loss, job duration, and job satisfaction.

Ascertaining whether people with VI face greater difficulties retaining employment is relevant given the importance of successful school-to-work transition with respect to future labor market participation. One potential future research approach could be to take salary changes into account as job changes are also associated with wage growth and may thus not necessarily be a disadvantage [32]. Additionally, an investigation into labor market prospects after completing further education may prove fruitful, by evaluating whether salary growth profiles associated with additional education are similar for people with VI. Moreover, there is a need to scrutinize legislative changes, evaluate policies, and find out what legal structures lead to the best possible outcomes for vulnerable groups that are at a disadvantage on the labor market. Another suggestion on how future research could expand the available research is by including other disability groups, such as deaf and hard-of-hearing people, or people with mental disabilities or illnesses, as comparison group. This would enrich our understanding of different challenges faced by different disability groups on the labor market. Lastly, a promising direction for further research may also be dynamic models to explore transitions between employment, unemployment and disability benefits over time. Such models would allow for a more nuanced understanding of the pathways individuals with visual impairments take over time and how these compare to those in the general population.

## Supplementary Information

Below is the link to the electronic supplementary material.

Supplementary Material 1

## Footnotes

1 By using a 1:1 nearest neighbor matching without replacement, we wanted to preserve interpretability within our competing risks framework. With mutually exclusive outcomes, using multiple controls per treated unit would require arbitrary aggregation of discrete outcomes. Additionally, given our large pool of potential control units, we were confident to retain balanced covariates without replacement.

2 To validate this re-matching approach, we have additionally conducted the analyses on a sample that keeps all the matches from the first matching model, and only if these matched counterparts do not both transition to employment, we replace them with the matched counterpart of the second matching method. Additionally, we have also run the duration models using only the matched pairs of the first matching procedures in which both obtained employment post-graduation. The resulting effect estimates do not differ significantly. Due to more precise estimates and greater confidence in conditional mean independence, the re-match model is our preferred choice. A table comparing the competing risks regression effect estimates is provided in the supplement, table s1.

3 See: https://www.government.nl/topics/health-insurance.

4 See: https://www.cbs.nl/nl-nl/nieuws/2016/27/vrijwel-alle-middelbare-scholieren-leren-door.

5 The estimated cost per recipient was calculated by dividing the total annual expenditure, based on the Verantwoordingsonderzoek Ministerie van Sociale Zaken en Werkgelegenheid 2024, by the number of recipients reported in CBS StatLine data; for our operationalization, social benefits combine the costs and recipient counts of bijstand, related income supplements, and Wajong. Ministerie van Sociale Zaken en Werkgelegenheid. Verantwoordingsonderzoek 2023 XV SZW. Algemene Rekenkamer (2024). https://www.rekenkamer.nl/publicaties/rapporten/2025/05/21/vo-2024-szw. Centraal Bureau voor de Statistiek. StatLine: Personen met bijstandsuitkering; kenmerken. (2024). https://opendata.cbs.nl/statline/#/CBS/nl/dataset/80578ned/table. Centraal Bureau voor de Statistiek. StatLine: Ontvangers arbeidsongeschiktheidsregelingen; aantallen. (2024). https://opendata.cbs.nl/statline/#/CBS/nl/dataset/85586NED/table.

6 A similar figure covering the first two years after graduation is available in the supplementary materials.

7 The plot in Fig. 3 is based on one observation per individual per status, when multiple secondary incomes were recorded, ties were resolved according to the hierarchy of outcomes maintained throughout this paper, (1) employment, (2) self-employment, (3) unemployment insurance, (4) social benefits, (5) illness/disability insurance, (6) attending further education. At status 1, there were 23 people (0.5%) with more than 1 secondary income, at status 2 there were 56 people (2.2%), with more than one secondary income.

8
https://www.uwv.nl/nl/ww/wanneer-recht-op-ww.
