# Supplementary material for: Labor market entry prospects of people with visual impairments: evidence from administrative register data in the Netherlands
Source: Eur J Health Econ. 2025 Sep 15;27(3):509–26. doi: 10.1007/s10198-025-01830-2 (PMC13190407; doi:10.1007/s10198-025-01830-2)

**Supplementary materials**

**Table 1** Comparison of competing risks regression effect estimates of visual impairment after the three different matching procedures

| Economic outcome à | (1) Employment | | (2) Self-employment | | (3) Unemployment insurance | | (4) Social benefits | | (5) Illness and disability insurance | | (6) education | | (7)  Other no income | |
| --- | --- | --- | --- | --- | --- | --- | --- | --- | --- | --- | --- | --- | --- | --- |
|  | B (SE) | p | B (SE) | p | B (SE) | p | B (SE) | P | B (SE) | p | B (SE) | p | B (SE) | p |
| *full re-match model* | 0.409 (0.069) | 0.000  *** | -0.613 (0.333) | 0.065 | 0.092 (0.164) | 0.580 | 1.662 (0.334) | 0.000 **** | 1.130 (0.245) | 0.000 *** | -0.156 (0.217) | 0.470 | -0.043 (0.113) | 0.700 |
| partial re-match mode | 0.423 (0.070) | 0.000 *** | -0.864 (0.312) | 0.056 | 0.041 (0.162) | 0.800 | 1.400 (0.309) | 0.000 *** | 1.300 (0.252) | 0.000 *** | 0.019 (0.223) | 0.930 | -0.063 (0.112) | 0.570 |
| first match with both employed | 0.436 (0.087) | 0.000 *** | -0.925 (0.401) | 0.021 | 0.039 (0.192) | 0.840 | 1.380 (0.351) | 0.000 ** * | 1.280 (0.295) | 0.000  ** * | 0.003 (0.296) | 0.990 | -0.080 (0.136) | 0.550 |
|  |  | |  | |  | |  | |  | |  | |  | |
| * p<0.05. ** p<0.01. *** p<0.001 |  | |  | |  | |  | |  | |  | |  | |

**Table 2**  Competing risks regression estimates for the school-to-work transition without age covariates

| Economic outcome 🡪 | (1) Employment | | (2) Self-employment | | (3) Unemployment insurance | | (4) Social benefits | | (5) Illness and disability insurance | | (6) education | |
| --- | --- | --- | --- | --- | --- | --- | --- | --- | --- | --- | --- | --- |
|  | B (SE) | p | B (SE) | p | B (SE) | p | B (SE) | P | B (SE) | p | B (SE) | p |
| *Visual impairment* | -0.388 (0.038) | 0.000  *** | -0.368 (0.170) | 0.030 * | -0.152 (0.279) | 0.590 | 2.001 (0.133) | 0.000 *** | 1.199 (0.202) | 0.000 ** | 0.110 (0.074) | 0.140 |
| Age at graduation | Not included | | Not included | | Not included | | Not included | | Not included | | Not included | |
| Higher order polynomials of age | Not included | | Not included | | Not included | | Not included | | Not included | | Not included | |
| Gender – woman | -0.025 (0.038) | 0.510 | 0.223 (0.169) | 0.190 | 0.204 (0.290) | 0.480 | -0.017 (0.092) | 0.850 | 0.188 (0.177) | 0.290 | 0.013 (0.075) | 0.870 |
| Migration – first generation | -0.289 (0.070) | 0.000 *** | -0.386 (0.335) | 0.250 | 0.551 (0.390) | 0.160 | 0.461 (0.140) | 0.000 *** | -0.563 (0.336) | 0.094 | 0.407 (0.133) | 0.002 ** |
| Migration – second generation | -0.423 (0.061) | 0.000 *** | 0.074 (0.255) | 0.770 | 0.396 (0.396) | 0.320 | 0.070 (0.131) | 0.590 | -0.439 (0.303) | 0.150 | 0.462 (0.091) | 0.000 *** |
| Education – middle | 0.218 (0.074) | 0.003 ** | 1.556 (0.584) | 0.008 ** | 1.402 (1.044) | 0.180 | -0.619 (0.142) | 0.000 *** | 1.178 (0.395) | 0.003 ** | 0.242 (0.130) | 0.063 |
| Education - high | 0.154 (0.072) | 0.033 * | 1.588 (0.588) | 0.007 | 2.165 (1.027) | 0.035 | -0.566 (0.144) | 0.000 *** | 0.514 (0.405) | 0.210 | -0.434 (0.149) | 0.004 |
| GPA | 0.029 (0.032) | 0.370 | 0.070 (0.129) | 0.000 *** | 0.156 (0.721) | 0.830 | 0.036 (0.090) | 0.690 | 0.113 (0.138) | 0.410 | -0.043 (0.039) | 0.270 |
| Birthyear | -0.012 (0.002) | 0.000 *** | -0.046 (0.005) | 0.000 | -0.012 (0.009) | 0.190 | 0.037 (0.004) | 0.000 *** | Not inc |  | 0.101 (0.007) | 0.000 *** |
| *N* | 4822 | | 4822 | | 4822 | | 4822 | | 4822 | | 4822 | |
|  |  | |  | |  | |  | |  | |  | |
| * p<0.05. ** p<0.01. *** p<0.001 |  | |  | |  | |  | |  | |  | |

**Table 3** competing risks regression estimates for school-to-work transition censoring distribution estimated per group

| Economic outcome 🡪 | (1) Employment | | (2) Self-employment | | (3) Unemployment insurance | | (4) Social benefits | | (5) Illness and disability insurance | | (6) education | |
| --- | --- | --- | --- | --- | --- | --- | --- | --- | --- | --- | --- | --- |
|  | B (SE) | p | B (SE) | p | B (SE) | p | B (SE) | P | B (SE) | p | B (SE) | p |
| *Visual impairment* | -0.406 (0.038) | 0.000  *** | -0.368 (0.170) | 0.030 * | -0.176 (0.280) | 0.530 | 2.028 (0.133) | 0.0000 *** | 0.593 (0.205) | 0.004 ** | 0.128 (0.074) | 0.083 |
| Age at graduation | 0.183 (0.012) | 0.000 *** | 0.082 (0.017) | 0.000 *** | -0.053 (0.032) | 0.097 | 0.346 (0.055) | 0.000 *** | 0.096 (0.027) | 0.001 ** | -0.246 (0.049) | 0.000 *** |
| Higher order polynomials of age | Included up to 2^nd^ order | | Not included | | Not included | | Not included | | Not included | | Included up to 2^nd^ order | |
| Gender – woman | -0.025 (0.038) | 0.520 | 0.244 (0.169) | 0.150 | 0.197 (0.288) | 0.490 | -0.011 (0.092) | 0.910 | -0.100 (0.180) | 0.580 | -0.006 (0.075) | 0.083 |
| Migration – first generation | -0.460 (0.073) | 0.000 *** | -0.413 (0.332) | 0.210 | 0.669 (0.396) | 0.091 | 0.318 (0.140) | 0.023 * | -0.219 (0.372) | 0.560 | 0.582 (0.136) | 0.000 *** |
| Migration – second generation | -0.442 (0.061) | 0.000 *** | 0.059 (0.256) | 0.820 | 0.423 (0.393) | 0.280 | 0.056 (0.128) | 0.660 | 0.280 (0.271) | 0.300 | 0.494 (0.091) | 0.000 *** |
| Education – middle | 0.280 (0.075) | 0.000 *** | 1.548 (0.583) | 0.008 ** | 1.184 (1.034) | 0.250 | -0.756 (0.144) | 0.000 *** | 0.642 (0.406) | 0.110 | 0.352 (0.132) | 0.008 ** |
| Education - high | 0.030 (0.075) | 0.690 | 1.756 (0.591) | 0.003 | 1.942 (1.037) | 0.061 | -0.965 (0.155) | 0.000 *** | 0.728 (0.405) | 0.072 | -0.125 (0.165) | 0.450 |
| GPA | 0.030 (0.033) | 0.360 | Not inc |  | Not inc |  | -1.009 (0.153) | 0.000 *** | 0.156 (0.155) | 0.320 | -0.047 (0.041) | 0.240 |
| Birthyear | -0.007 (0.005) | 0.180 | 0.032 (0.016) | 0.050 | -0.071 (0.027) | 0.009 ** | 0.030 (0.055) | 0.000 *** | -0.029 (0.026) | 0.260 | 0.047 (0.010) | 0.000 *** |
| *N* | 4822 | | 4822 | | 4822 | | 4822 | | 4822 | | 4822 | |
| $R^{2}$ |  | |  | |  | |  | |  | |  | |
|  |  | |  | |  | |  | |  | |  | |
| * p<0.05. ** p<0.01. *** p<0.001 |  | |  | |  | |  | |  | |  | |

**Figure 1** Transitions into socio economic outcomes throughout first two years after graduation


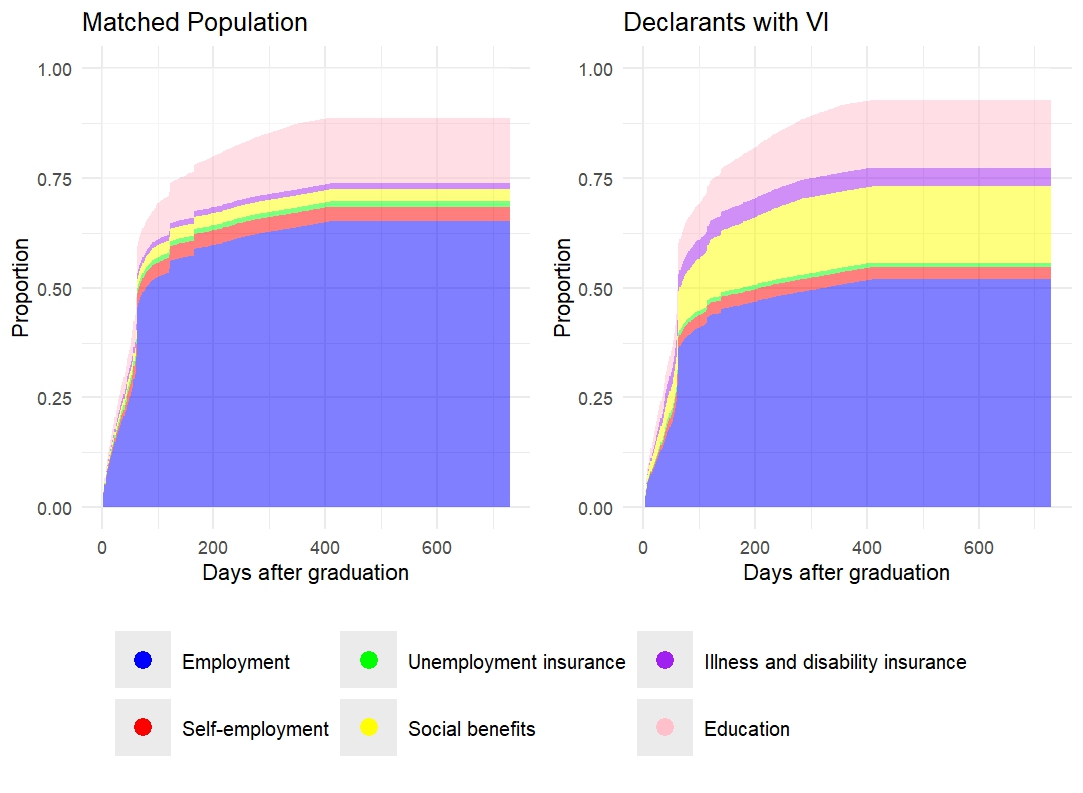


**Table 4** Results of the competing risks regression analysis post-employment entry without age covariates

| Economic outcome 🡪 | (1) Employment | | (2) Self-employment | | (3) Unemployment insurance | | (4) Social benefits | | (5) Illness and disability insurance | | (6) education | | (7) other no income | |
| --- | --- | --- | --- | --- | --- | --- | --- | --- | --- | --- | --- | --- | --- | --- |
|  | B (SE) | p | B (SE) | p | B (SE) | p | B (SE) | P | B (SE) | p | B (SE) | p | B (SE) | P |
| *Visual impairment* | 0.391 (0.064) | 0.000 *** | -0.547 (0.330) | 0.097 | 0.060 (0.165) | 0.710 | 1.649 (0.335) | 0.000 *** | 1.119 (0.244) | 0.000 *** | -0.250 (0.210) | 0.230 | -0.140 (0.113) | 0.210 |
| Age at first job entrance | Not included | | Not included | | Not included | | Not included | | Not included | | Not included | | Not included | |
| Higher order polynomials of age | Not included | | Not included | | Not included | | Not included | | Not included | | Not included | | Not included | |
| Gender – woman | 0.179 (0.068) | 0.009 * | 0.314 (0.338) | 0.350 | -0.348 (0.162) | 0.032 * | -0.574 (0.264) | 0.030 * | 0.430 (0.196) | 0.028 | -0.276 (0.215) | 0.200 | 0.020 (0.115) | 0.860 |
| Migration – first generation | -0.061 (0.122) | 0.620 | Not included |  | 0.384 (0.249) | 0.120 | -0.148 (0.465) | 0.750 | -0.125 (0.336) | 0.710 | 0.658 (0.328) | 0.045 * | 0.319 (0.191) | 0.096 |
| Migration – second generation | -0.003 (0.117) | 0.980 | Not included |  | -0.107 (0.285) | 0.710 | -0.471 (0.518) | 0.360 | 0.299 (0.313) | 0.340 | 0.044 (0.325) | 0.890 | 0.139 (0.175) | 0.430 |
| Education – middle | -0.248 (0.117) | 0.018* | Not included | | 0.465 (0.332) | 0.160 | -0.386 (0.375) | 0.300 | 0.295 (0.326) | 0.370 | -0.109 (0.342) | 0.750 | -0.399 (0.202) | 0.048 * |
| Education - high | -0.148 (0.103) | 0.150 | 1.014 (0.345) | 0.003 | 0.569 (0.323) | 0.078 | -0.531 (0.391) | 0.170 | 0.073 (0.332) | 0.830 | -0.838 (0.386) | 0.030 * | 0.092 (0.196) | 0.640 |
| GPA | -0.059 (0.075) | 0.430 | Not included |  | -0.238 (0.106) | 0.025 | 0.169 (0.293) | 0.560 | 0.315 (0.469) | 0.500 | 0.082 (0.102) | 0.420 | -0.117 (0.104) | 0.260 |
| Birthyear | -0.005 (0.003) | 0.071 | -0.023 (0.012) | 0.053 | 0.004 (0.007) | 0.570 | 0.015 (0.010) | 0.140 | -0.049 (0.009) | 0.000 *** | 0.095 (0.017) | 0.000 *** | 0.036 (0.006) | 0.000 *** |
| *N* | 2504 | | 2504 | | 2504 | | 2504 | | 2504 | | 2504 | | 2504 | |
|  |  | |  | |  | |  | |  | |  | |  | |
| * p<0.05. ** p<0.01. *** p<0.001 |  | |  | |  | |  | |  | |  | |  | |

**Table 5** competing risks regression estimates after initial employment entry censoring distribution estimated per group

| Economic outcome 🡪 | (1) Employment | | (2) Self-employment | | (3) Unemployment insurance | | (4) Social benefits | | (5) Illness and disability insurance | | (6) education | | (7) other no income | |
| --- | --- | --- | --- | --- | --- | --- | --- | --- | --- | --- | --- | --- | --- | --- |
|  | B (SE) | p | B (SE) | p | B (SE) | p | B (SE) | P | B (SE) | p | B (SE) | p | B (SE) | P |
| *Visual impairment* | 0.409 (0.069) | 0.000*** | -0.613 (0.333) | 0.065 | 0.092 (0.164) | 0.580 | 1.662 (0.334) | 0.000 *** | 1.130 (0.245) | 0.000 *** | -0.156 (0.217) | 0.470 | -0.043 (0.113) | 0.700 |
| Age at first job entrance | -0.042 (0.025) | 0.099 | 0.224 (0.124) | 0.071 | 0.219 (0.338) | 0.520 | 0.288 (0.370) | 0.440 | 0.104 (0.300) | 0.730 | -0.609 (0.434) | 0.160 | 0.026 (0.197) | 0.900 |
| Higher order polynomials of age | Included up to 2^nd^ order | | Included up to 2nd order | | Included up to 3^rd^ order | | Included up to 3^rd^ order | | Included up to 3^rd^ order | | Included up to 3^rd^ order | | Included up to 3^rd^ order | |
| Gender – woman | 0.160 (0.068) | 0.018 * | 0.331 (0.333) | 0.320 | -0.367 (0.161) | 0.023 * | -0.607 (0.263) | 0.021 * | 0.488 (0.200) | 0.140 | 0.269 (0.223) | 0.230 | -0.036 (0.115) | 0.760 |
| Migration – first generation | -0.050 (0.123) | 0.690 | -0.606 (0.733) | 0.410 | 0.367 (0.248) | 0.140 | -0.239 (0.468) | 0.610 | -0.060 (0.336) | 0.860 | 1.030 (0.331) | 0.002** | 0.495 (0.195) | 0.011 * |
| Migration – second generation | -0.012 (0.118) | 0.920 | 0.420 (0.445) | 0.350 | -0.130 (0.282) | 0.640 | -0.517 (0.522) | 0.320 | 0.334 (0.312) | 0.280 | 1.199 (0.330) | 0.550 | 0.146 (0.175) | 0.400 |
| Education – middle | -0.267 (0.105) | 0.011* | Not included | | 0.436 (0.335) | 0.190 | -0.374 (0.386) | 0.330 | 0.249 (0.324) | 0.440 | 0.348 (0.342) | 0.920 | -0.522 (0.206) | 0.011 |
| Education - high | -0.250 (0.106) | 0.018* | 0.897 (0.338) | 0.008 | 0.314 (0.328) | 0.340 | -0.766 (0.409) | 0.061 | 0.180 (0.346) | 0.600 | -0.254 (0.441) | 0.570 | -0.163 (0.212) | 0.440 |
| GPA | -0.058 (0.078) | 0.460 | 0.159 (0.303) | 0.600 | -0.244 (0.110) | 0.027 | 0.184 (0.324) | 0.570 | 0.302 (0.449) | 0.500 | 0.093 (0.109) | 0.400 | -0.107 (0.117) | 0.360 |
| Birthyear | -0.044 (0.008) | 0.000 *** | -0.035 (0.029) | 0.230 | -0.057 (0.020) | 0.006 ** | -0.028 (0.028) | 0.310 | 0.008 (0.024) | 0.750 | 0.0.004 (0.030) | 0.910 | -0.062 (0.014) | 0.000 *** |
| *N* | 2504 | | 2504 | | 2504 | | 2504 | | 2504 | | 2504 | | 2504 | |
| $R^{2}$ | 0 | |  | |  | |  | |  | |  | |  | |
|  |  | |  | |  | |  | |  | |  | |  | |
| * p<0.05. ** p<0.01. *** p<0.001 |  | |  | |  | |  | |  | |  | |  | |

**Figure 2** Dynamics into socio economic categories throughout the first 2 years post first employment


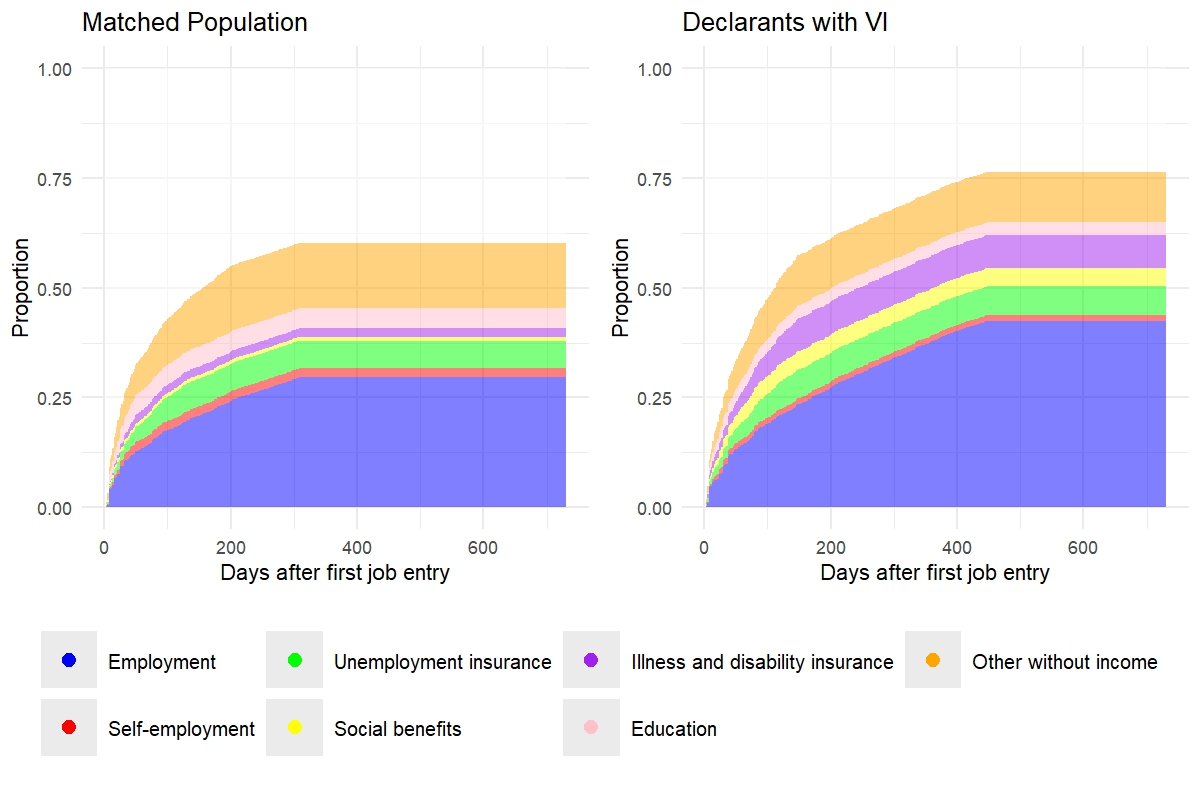

Supplement: Supplementary file 1 — Supplementary Material 1 [file 10198_2025_1830_MOESM1_ESM.docx]
